# 2,2-Dichloro-*N*-(4-methyl­phenyl­sulfonyl)acetamide

**DOI:** 10.1107/S160053680802134X

**Published:** 2008-07-16

**Authors:** B. Thimme Gowda, Sabine Foro, P. G. Nirmala, B. P. Sowmya, Hartmut Fuess

**Affiliations:** a Department of Chemistry, Mangalore University, Mangalagangotri 574 199, Mangalore, India; b Institute of Materials Science, Darmstadt University of Technology, Petersenstrasse 23, D-64287 Darmstadt, Germany

## Abstract

The N—H and C=O bonds in the title compound, C_9_H_9_Cl_2_NO_3_S, are *trans* to each other, similar to what is observed in 2,2,2-trimethyl-*N*-(phenyl­sulfon­yl)acetamide and 2,2,2-trimethyl-*N*-(4-methyl­phenyl­sulfon­yl)acetamide. The bond parameters in the title compound are also similar to those in the aforementioned two structures. N—H⋯O hydrogen bonds connect the mol­ecules into chains running along the *a* axis.

## Related literature

For related literature, see: Gowda *et al.* (2006 ▶, 2007 ▶, 2008*a* ▶,*b* ▶).
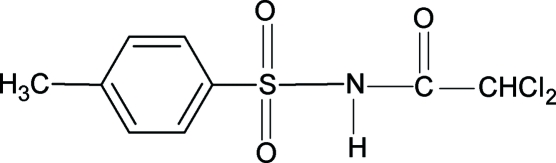


## Experimental

### 

#### Crystal data

C_9_H_9_Cl_2_NO_3_S
*M* *_r_* = 282.13
Orthorhombic, 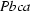

*a* = 9.6580 (8) Å
*b* = 10.3177 (8) Å
*c* = 23.067 (2) Å
*V* = 2298.6 (3) Å^3^
*Z* = 8
Mo *K*α radiation
μ = 0.74 mm^−1^
*T* = 299 (2) K
0.48 × 0.46 × 0.32 mm

#### Data collection

Oxford Diffraction Xcalibur diffractometer with a Sapphire CCD detector
Absorption correction: multi-scan (*CrysAlis RED*; Oxford Diffraction, 2007 ▶) *T*_min_ = 0.719, *T*_max_ = 0.799
8079 measured reflections
2313 independent reflections
1886 reflections with *I* > 2σ(*I*)
*R*_int_ = 0.022

#### Refinement

*R*[*F* ^2^ > 2σ(*F* ^2^)] = 0.034
*wR*(*F* ^2^) = 0.099
*S* = 1.14
2313 reflections
150 parameters
H atoms treated by a mixture of independent and constrained refinement
Δρ_max_ = 0.34 e Å^−3^
Δρ_min_ = −0.33 e Å^−3^

### 

Data collection: *CrysAlis CCD* (Oxford Diffraction, 2004 ▶); cell refinement: *CrysAlis RED* (Oxford Diffraction, 2007 ▶); data reduction: *CrysAlis RED*; program(s) used to solve structure: *SHELXS97* (Sheldrick, 2008 ▶); program(s) used to refine structure: *SHELXL97* (Sheldrick, 2008 ▶); molecular graphics: *PLATON* (Spek, 2003 ▶); software used to prepare material for publication: *SHELXL97*.

## Supplementary Material

Crystal structure: contains datablocks I, global. DOI: 10.1107/S160053680802134X/bt2744sup1.cif

Structure factors: contains datablocks I. DOI: 10.1107/S160053680802134X/bt2744Isup2.hkl

Additional supplementary materials:  crystallographic information; 3D view; checkCIF report

## Figures and Tables

**Table 1:** Hydrogen-bond geometry (Å, °)

*D*—H⋯*A*	*D*—H	H⋯*A*	*D*⋯*A*	*D*—H⋯*A*
N1—H1N⋯O3^i^	0.82 (3)	2.03 (3)	2.833 (2)	166 (2)

Symmetry code: (i) 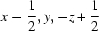.

**Fig. 1.**
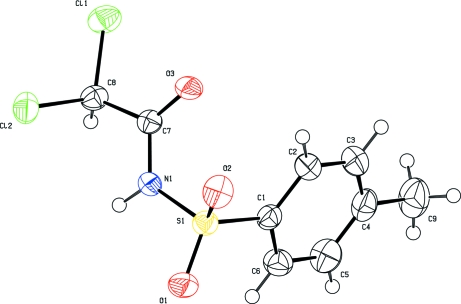
Molecular structure of (I), showing the atom labeling scheme. The displacement ellipsoids are drawn at the 50% probability level. The H atoms are represented as small spheres of arbitrary radii.

**Fig. 2.**
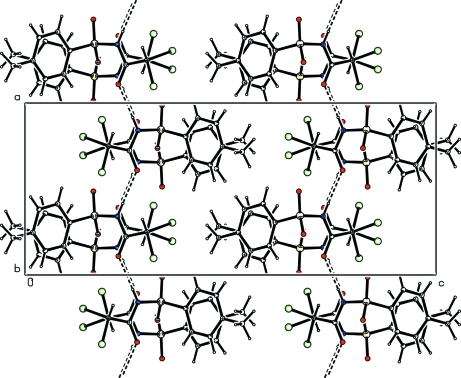
Molecular packing of (I) with hydrogen bonding shown as dashed lines.

## supplementary crystallographic information

### Comment

As part of a study of the substituent effects on the solid state geometries of *N*-(aryl)-sulfonamides and substituted amides, the structure of *N*-(4-methylphenylsulfonyl)-2,2-dichloroacetamide (N4MPSDCAA)   has been determined (Gowda *et al.*, 2006, 2007, 2008*a*, 2008*b*). The conformation  of the N—H and C=O bonds   in N4MPSDCAA are anti to each other (Fig. 1), similar to that observed in *N*-(phenylsulfonyl)-2,2,2-trimethylacetamide (NPSTMAA) (Gowda *et al.*, 2008*b*), *N*-(4-chlorophenylsulfonyl)-2,2,2-trimethylacetamide (N4CPSTMAA) and (4-methylphenylsulfonyl)-2,2,2-trimethylacetamide (N4MPSTMAA) (Gowda *et al.*, 2008*a, b*). The bond parameters in N4MPSDCAA are similar to those in NPSTMAA, N4MPSTMAA, N4CPSTMAA (Gowda *et al.*, 2008*a, b*), *N*-(aryl)-2,2-dichloroacetamides (Gowda *et al.*, 2006) and 4-methylbenzenesulfonamide and other arylsulfonamides (Gowda *et al.*, 2007). The N—H···O hydrogen bonds (Table 1) connect the molecules to  chains running along the a axis (Fig. 2).

### Experimental

The title compound was prepared by refluxing 4-methylbenzenesulfonamide (0.10 mole) with an excess dichloroacetyl chloride (0.20 mole) for about an hour on a water bath. The reaction mixture was cooled and poured into ice cold water. The resulting solid was separated, washed thoroughly with water and dissolved in warm dilute sodium hydrogen carbonate solution. The title compound was precipitated by acidifying the filtered solution with glacial acetic acid. It was filtered, dried and recrystallized from ethanol. The purity of the compound was checked by determining its melting point. It was characterized by recording its infrared and NMR spectra. Single crystals of the title compound used for X-ray diffraction studies were obtained from a slow evaporation of an ethanolic solution.

### Refinement

The NH atom was located in difference map, and its positional parameters were refined freely. The other H atoms were positioned with idealized geometry using a riding model with C—H = 0.93–0.98 Å. All H atoms were refined with isotropic displacement parameters (set to 1.2 times of the *U*_eq_ of the parent atom).

The five reflections most deviating reflecions (1 0 2, 1 1 2, 0 2 0, 1 1 1, 0 0 4) were omitted from the refinement.

### Crystal data

**Table:**

C_9_H_9_Cl_2_NO_3_S	*F*_000_ = 1152
*M**_r_* = 282.13	*D*_x_ = 1.631 Mg m^−^^3^
Orthorhombic, *P**b**c**a*	Mo *K*α radiation λ = 0.71073 Å
Hall symbol: -P 2ac 2ab	Cell parameters from 3825 reflections
*a* = 9.6580 (8) Å	θ = 2.3–28.0º
*b* = 10.3177 (8) Å	µ = 0.74 mm^−^^1^
*c* = 23.067 (2) Å	*T* = 299 (2) K
*V* = 2298.6 (3) Å^3^	Prism, colourless
*Z* = 8	0.48 × 0.46 × 0.32 mm

### Data collection

**Table:**

Oxford Diffraction Xcalibur diffractometer with a Sapphire CCD detector	2313 independent reflections
Radiation source: fine-focus sealed tube	1886 reflections with *I* > 2σ(*I*)
Monochromator: graphite	*R*_int_ = 0.022
*T* = 299(2) K	θ_max_ = 26.4º
Rotation method data acquisition using ω and φ scans	θ_min_ = 3.9º
Absorption correction: multi-scan(CrysAlis RED; Oxford Diffraction, 2007)	*h* = −12→11
*T*_min_ = 0.719, *T*_max_ = 0.799	*k* = −12→11
8079 measured reflections	*l* = −28→28

### Refinement

**Table:**

Refinement on *F*^2^	Hydrogen site location: inferred from neighbouring sites
Least-squares matrix: full	H atoms treated by a mixture of independent and constrained refinement
*R*[*F*^2^ > 2σ(*F*^2^)] = 0.034	*w* = 1/[σ^2^(*F*_o_^2^) + (0.0465*P*)^2^ + 1.5357*P*] where *P* = (*F*_o_^2^ + 2*F*_c_^2^)/3
*wR*(*F*^2^) = 0.099	(Δ/σ)_max_ = 0.001
*S* = 1.14	Δρ_max_ = 0.34 e Å^−^^3^
2313 reflections	Δρ_min_ = −0.33 e Å^−^^3^
150 parameters	Extinction correction: SHELXL97 (Sheldrick, 2008), Fc^*^=kFc[1+0.001xFc^2^λ^3^/sin(2θ)]^-1/4^
Primary atom site location: structure-invariant direct methods	Extinction coefficient: 0.0216 (12)
Secondary atom site location: difference Fourier map	

### Special details

**Table:**

Experimental. CrysAlis RED (Oxford Diffraction, 2007) Empirical absorption correction using spherical harmonics, implemented in SCALE3 ABSPACK scaling algorithm.
Geometry. All e.s.d.'s (except the e.s.d. in the dihedral angle between two l.s. planes) are estimated using the full covariance matrix. The cell e.s.d.'s are taken into account individually in the estimation of e.s.d.'s in distances, angles and torsion angles; correlations between e.s.d.'s in cell parameters are only used when they are defined by crystal symmetry. An approximate (isotropic) treatment of cell e.s.d.'s is used for estimating e.s.d.'s involving l.s. planes.
Refinement. Refinement of *F*^2^ against ALL reflections. The weighted *R*-factor *wR* and goodness of fit *S* are based on *F*^2^, conventional *R*-factors *R* are based on *F*, with *F* set to zero for negative *F*^2^. The threshold expression of *F*^2^ > σ(*F*^2^) is used only for calculating *R*-factors(gt) *etc*. and is not relevant to the choice of reflections for refinement. *R*-factors based on *F*^2^ are statistically about twice as large as those based on *F*, and *R*- factors based on ALL data will be even larger.

### Fractional atomic coordinates and isotropic or equivalent isotropic displacement parameters (Å^2^)

**Table:**

	*x*	*y*	*z*	*U*_iso_*/*U*_eq_	
C1	0.1940 (2)	0.2089 (2)	0.10903 (9)	0.0321 (5)	
C2	0.3304 (3)	0.2325 (3)	0.09411 (11)	0.0419 (6)	
H2	0.4022	0.1916	0.1138	0.050*	
C3	0.3581 (3)	0.3181 (3)	0.04945 (11)	0.0487 (6)	
H3	0.4497	0.3348	0.0395	0.058*	
C4	0.2532 (3)	0.3796 (2)	0.01912 (10)	0.0440 (6)	
C5	0.1181 (3)	0.3514 (3)	0.03428 (12)	0.0480 (6)	
H5	0.0463	0.3897	0.0136	0.058*	
C6	0.0866 (3)	0.2678 (2)	0.07928 (11)	0.0403 (6)	
H6	−0.0050	0.2515	0.0893	0.048*	
C7	0.2763 (2)	0.27000 (19)	0.24611 (9)	0.0279 (4)	
C8	0.2502 (2)	0.3642 (2)	0.29677 (9)	0.0331 (5)	
H8	0.1773	0.4255	0.2859	0.040*	
C9	0.2837 (4)	0.4760 (3)	−0.02853 (13)	0.0626 (8)	
H9A	0.3703	0.4545	−0.0466	0.075*	
H9B	0.2892	0.5616	−0.0124	0.075*	
H9C	0.2111	0.4731	−0.0569	0.075*	
N1	0.15881 (18)	0.21543 (18)	0.22498 (8)	0.0292 (4)	
H1N	0.084 (3)	0.238 (2)	0.2380 (11)	0.035*	
O1	0.01360 (18)	0.07170 (18)	0.16642 (7)	0.0438 (4)	
O2	0.26226 (19)	0.01713 (16)	0.17765 (7)	0.0433 (4)	
O3	0.39079 (15)	0.24827 (17)	0.22726 (7)	0.0385 (4)	
Cl1	0.40283 (7)	0.45024 (6)	0.31278 (3)	0.0454 (2)	
Cl2	0.19595 (7)	0.27418 (7)	0.35847 (3)	0.0508 (2)	
S1	0.15508 (6)	0.11055 (5)	0.16929 (2)	0.03130 (18)	

### Atomic displacement parameters (Å^2^)

**Table:**

	*U*^11^	*U*^22^	*U*^33^	*U*^12^	*U*^13^	*U*^23^
C1	0.0352 (11)	0.0326 (11)	0.0285 (10)	−0.0022 (9)	−0.0002 (8)	−0.0039 (9)
C2	0.0335 (12)	0.0556 (16)	0.0366 (12)	−0.0025 (11)	0.0009 (10)	0.0018 (11)
C3	0.0431 (14)	0.0605 (17)	0.0424 (13)	−0.0122 (12)	0.0083 (11)	0.0013 (13)
C4	0.0633 (16)	0.0372 (13)	0.0315 (11)	−0.0068 (11)	0.0046 (11)	−0.0024 (10)
C5	0.0556 (16)	0.0421 (14)	0.0463 (14)	0.0060 (12)	−0.0039 (12)	0.0077 (12)
C6	0.0353 (12)	0.0431 (14)	0.0424 (13)	0.0005 (10)	−0.0013 (10)	0.0029 (11)
C7	0.0282 (11)	0.0269 (10)	0.0285 (10)	0.0026 (8)	−0.0035 (8)	0.0052 (8)
C8	0.0323 (11)	0.0314 (11)	0.0355 (11)	0.0042 (9)	−0.0043 (9)	−0.0018 (9)
C9	0.087 (2)	0.0531 (18)	0.0476 (15)	−0.0120 (16)	0.0097 (15)	0.0089 (13)
N1	0.0233 (9)	0.0331 (10)	0.0312 (9)	0.0009 (7)	0.0022 (7)	−0.0022 (8)
O1	0.0407 (10)	0.0470 (10)	0.0436 (9)	−0.0165 (8)	−0.0027 (7)	−0.0009 (8)
O2	0.0524 (10)	0.0314 (9)	0.0462 (9)	0.0083 (8)	0.0008 (8)	−0.0002 (7)
O3	0.0230 (8)	0.0514 (10)	0.0412 (9)	0.0030 (7)	−0.0015 (6)	−0.0083 (7)
Cl1	0.0513 (4)	0.0353 (3)	0.0496 (4)	−0.0104 (3)	−0.0075 (3)	−0.0046 (3)
Cl2	0.0564 (4)	0.0610 (4)	0.0348 (3)	−0.0153 (3)	0.0085 (3)	−0.0048 (3)
S1	0.0327 (3)	0.0290 (3)	0.0322 (3)	−0.0035 (2)	−0.0011 (2)	−0.0005 (2)

### Geometric parameters (Å, °)

**Table:**

C1—C2	1.384 (3)	C7—N1	1.357 (3)
C1—C6	1.384 (3)	C7—C8	1.540 (3)
C1—S1	1.761 (2)	C8—Cl1	1.760 (2)
C2—C3	1.383 (4)	C8—Cl2	1.778 (2)
C2—H2	0.9300	C8—H8	0.9800
C3—C4	1.385 (4)	C9—H9A	0.9600
C3—H3	0.9300	C9—H9B	0.9600
C4—C5	1.381 (4)	C9—H9C	0.9600
C4—C9	1.512 (4)	N1—S1	1.6800 (19)
C5—C6	1.384 (4)	N1—H1N	0.82 (3)
C5—H5	0.9300	O1—S1	1.4256 (17)
C6—H6	0.9300	O2—S1	1.4276 (17)
C7—O3	1.210 (3)		
			
C2—C1—C6	120.8 (2)	C7—C8—Cl1	109.94 (15)
C2—C1—S1	120.07 (18)	C7—C8—Cl2	109.02 (14)
C6—C1—S1	118.98 (18)	Cl1—C8—Cl2	110.02 (12)
C3—C2—C1	118.8 (2)	C7—C8—H8	109.3
C3—C2—H2	120.6	Cl1—C8—H8	109.3
C1—C2—H2	120.6	Cl2—C8—H8	109.3
C2—C3—C4	121.8 (2)	C4—C9—H9A	109.5
C2—C3—H3	119.1	C4—C9—H9B	109.5
C4—C3—H3	119.1	H9A—C9—H9B	109.5
C5—C4—C3	117.8 (2)	C4—C9—H9C	109.5
C5—C4—C9	120.5 (3)	H9A—C9—H9C	109.5
C3—C4—C9	121.7 (3)	H9B—C9—H9C	109.5
C4—C5—C6	121.9 (2)	C7—N1—S1	124.04 (15)
C4—C5—H5	119.0	C7—N1—H1N	119.4 (18)
C6—C5—H5	119.0	S1—N1—H1N	116.4 (18)
C5—C6—C1	118.7 (2)	O1—S1—O2	120.76 (11)
C5—C6—H6	120.6	O1—S1—N1	103.72 (10)
C1—C6—H6	120.6	O2—S1—N1	108.43 (10)
O3—C7—N1	124.0 (2)	O1—S1—C1	109.25 (11)
O3—C7—C8	122.60 (19)	O2—S1—C1	109.93 (11)
N1—C7—C8	113.45 (18)	N1—S1—C1	103.17 (10)
			
C6—C1—C2—C3	1.1 (4)	N1—C7—C8—Cl2	69.4 (2)
S1—C1—C2—C3	−175.0 (2)	O3—C7—N1—S1	−0.4 (3)
C1—C2—C3—C4	−0.5 (4)	C8—C7—N1—S1	179.19 (14)
C2—C3—C4—C5	−0.9 (4)	C7—N1—S1—O1	174.97 (17)
C2—C3—C4—C9	178.2 (3)	C7—N1—S1—O2	45.5 (2)
C3—C4—C5—C6	1.9 (4)	C7—N1—S1—C1	−71.09 (19)
C9—C4—C5—C6	−177.2 (3)	C2—C1—S1—O1	−164.08 (19)
C4—C5—C6—C1	−1.4 (4)	C6—C1—S1—O1	19.7 (2)
C2—C1—C6—C5	−0.2 (4)	C2—C1—S1—O2	−29.4 (2)
S1—C1—C6—C5	175.97 (19)	C6—C1—S1—O2	154.39 (18)
O3—C7—C8—Cl1	9.7 (3)	C2—C1—S1—N1	86.1 (2)
N1—C7—C8—Cl1	−169.93 (15)	C6—C1—S1—N1	−90.13 (19)
O3—C7—C8—Cl2	−111.0 (2)		

### Hydrogen-bond geometry (Å, °)

**Table:**

*D*—H···*A*	*D*—H	H···*A*	*D*···*A*	*D*—H···*A*
N1—H1N···O3^i^	0.82 (3)	2.03 (3)	2.833 (2)	166 (2)

Symmetry codes: (i) *x*−1/2, *y*, −*z*+1/2.
